# A Mobile-Based Intervention for Dietary Behavior and Physical Activity Change in Individuals at High Risk for Type 2 Diabetes Mellitus: Randomized Controlled Trial

**DOI:** 10.2196/19869

**Published:** 2020-11-03

**Authors:** Zidu Xu, Ji Geng, Shuai Zhang, Kexin Zhang, Lin Yang, Jing Li, Jiao Li

**Affiliations:** 1 Institute of Medical Information and Library Chinese Academy of Medical Sciences and Peking Union Medical College Beijing China; 2 School of Nursing Peking Union Medical College Beijing China; 3 Nursing Department Peking Union Medical College Hospital Chinese Academy of Medical Sciences and Peking Union Medical College Beijing China

**Keywords:** transtheoretical model, type 2 diabetes mellitus, high risk, social media, dietary behavior, physical activity

## Abstract

**Background:**

Intensive lifestyle modifications have proved effective in preventing type 2 diabetes mellitus (T2DM), yet the efficiency and effectiveness of these modifications need to be improved. Emerging social media interventions are considered useful in promoting these lifestyles; nevertheless, few studies have investigated the effectiveness of combining them with behavior theory.

**Objective:**

This study aims to examine the effectiveness of a 6-month mobile-based intervention (DHealthBar, a WeChat applet) combined with behavioral theory compared with a printed intervention in improving dietary behaviors, physical activity, and intention to change these behaviors among populations at high risk for T2DM.

**Methods:**

Participants aged 23 to 67 years were recruited offline in Beijing, China, and were randomized into the intervention group or the control group, which received educational content via DHealthBar or a printed handbook, respectively. Educational materials were culturally tailored recommendations on improving dietary behaviors, physical activity, and intention to change based on the transtheoretical model. Participants in the intervention arm received push notifications twice per week on WeChat and had access to the educational content for the 6-month study period. Participants in the control arm received the same intervention content through printed materials. The outcomes of participants’ behavior change, intention to change behavior, and anthropometric characteristics were collected via online measuring tools at baseline, 3 months, and 6 months.

**Results:**

In this study, 79 enrolled individuals completed baseline information collection (control: n=38 vs intervention: n=41), and 96% (76/79) completed the 6-month follow-up visit. Attrition rates did not differ significantly between the 2 groups (*χ*^2^_1_=0.0, *P*=.61). Baseline equivalence was found. Participants in both groups reported a statistically significant decrease in energy intake at the 2 follow-up assessments compared with baseline (3 months, control: exp[**β**]=0.83, 95% CI 0.74-0.92 vs intervention: exp[**β**]=0.76, 95% CI 0.68-0.85; 6 months, control: exp[**β**]=0.87, 95% CI 0.78-0.96 vs intervention: exp[**β**]=0.57, 95% CI 0.51-0.64). At 6 months, a significantly larger decrease was observed in the intervention group in energy, fat, and carbohydrate intake, accompanied with a significantly larger increase in moderate-intensity physical activity compared with the control group (energy: exp[**β**]=0.66, 95% CI 0.56-0.77; fat: exp[**β**]=0.71, 95% CI 0.54-0.95; carbohydrates: exp[**β**]=0.83, 95% CI 0.66-1.03; moderate-intensity physical activity: exp[**β**]=2.05, 95% CI 1.23-3.44). After 6 months of the intervention, participants in the intervention group were more likely to be at higher stages of dietary behaviors (exp[**β**]=26.80, 95% CI 3.51-204.91) and physical activity (exp[**β**]=15.60, 95% CI 2.67-91.04) than the control group.

**Conclusions:**

DHealthBar was initially effective in improving dietary behavior, physical activity, and intention to change these behaviors among populations who were at high risk of developing T2DM, with significant differences in the changes of outcomes over the 6-month intervention period.

**Trial Registration:**

Chinese Clinical Trial Registry ChiCTR2000032323; https://tinyurl.com/y4h8q4uf

## Introduction

Type 2 diabetes mellitus (T2DM) accounts for over 90% of diabetes mellitus cases [1]. According to the International Diabetes Federation [2], China has the highest number of diabetic adults, with 1 in 10 adults aged 20 to 79 years (116 million adults) having diabetes mellitus. Unhealthy lifestyles are responsible for the increased risk of T2DM, including habitual sedentary behavior, overindulgence in animal fat, refined carbohydrates, and sugar-sweetened beverages [3-5]. Medication interventions, such as metformin, have been recommended as a preventive measure; nevertheless, for long-term effects, lifestyle interventions provide better performance, as described in follow-up studies such as the US Diabetes Prevention Program (DPP) and the Da Qing Study [6,7]. The 30-year Da Qing follow-up study reported that a median delay in diabetes onset of 3.96 years and a reduction in diabetes incidence and its complications were achieved, with an additional 1.44-year extension in life expectancy in the intervention group [6]. However, despite these promising results in curbing the diabetes epidemic, the typical face-to-face approaches of behavioral interventions might have trouble reaching a wider population to achieve and sustain healthy lifestyles, especially considering the high cost of current procedures (ie, physician visits, nutrition services, personal consultants, etc) [7].

With advanced information and communication technology and ubiquitous smartphones, medical services based on mobile phones and social networking services have been suggested as promising alternatives for cost-effective delivery of scalable behavior change interventions [8]. Trials have shown the potential of social media (eg, WeChat, Facebook, and Twitter) in delivering behavioral interventions on weight loss, diet pattern reconstruction, and online exercise coaching [9-11]. WeChat, a Chinese multipurpose social media app with a wide range of functions, had nearly 1.151 billion monthly active users on the service platform and over 300 million daily active users of extending applets in the third quarter of 2019, making it well suited to disseminate mobile health (mHealth) interventions in China [12,13]. However, the explosion of mHealth interventions and services does not mean that these interventions are ready for wide use. Rather, they have yet to be sufficiently explored and evaluated. If mHealth interventions were purely self-guided, a lot of information would overload the individuals and deter their adherence [14]. Participants might end up not using the intervention, resulting in low response.

Investigators have called for incorporating behavior theory to nudge mHealth interventions to be more effective in promoting health behaviors [15]. The transtheoretical model (TTM) has long been the most widely accepted model of health behavior change [16]. TTM focuses on the intention of individuals and assumes that the decision making and implementation of behavior change is a continuous, cyclical process divided into precontemplation, contemplation, preparation, action, and maintenance [17]. It has been suggested that TTM performs well in measuring one's current stage of behavior change and accounting for the dynamic process over time [18,19], thus making it possible to provide tailored interventions for individuals at various stages of health behavior change. The individuals would therefore be propelled toward higher stages of behavior change.

TTM-based behavior interventions have been proven effective [18-21]. Lee et al [22] noted that TTM-based interventions have been applied to physical activity and diet improvement among adolescents and male workers using web-based delivery methods. TTM-based interventions have also been successful in lifestyle management among patients with diabetes [22,23]. However, few studies have combined social media delivery modes with TTM-based strategies in tailored health behavior interventions, let alone applied them to high-risk populations for T2DM. In this context, we focused on high-risk individuals and used TTM-based interventions delivered through social media to expand the delivery modes and elements of the TTM-based interventions.

The aim of this study was to investigate the effectiveness of a 6-month WeChat-based (mobile-based) behavior intervention driven by TTM compared with a print-based intervention in promoting dietary behaviors and physical activity among individuals at high risk for T2DM. The secondary purpose was to compare changes in participants’ intentions to change these behaviors. Changes in anthropometric characteristics (ie, BMI and waist circumference) were also considered.

## Methods

### Research Design

The effectiveness of the TTM-based social media intervention was examined with a 2-arm randomized controlled trial registered on the Chinese Clinical Trial Registry (ChiCTR2000032323). Under the condition of receiving a written and oral explanation of the program requirements and giving informed consent, participants were randomly allocated to either (1) the DHealthBar intervention group or (2) the control group, which received printed materials. The control group was set to obtain stand-alone information about the effect of theory-driven interventions in improving health behaviors [18-20]. All study protocols, the consent process, and participant communications were approved by the School of Nursing, Peking Union Medical College institutional review board for the protection of human subjects (202002). See Multimedia Appendix 1 for the CONSORT-EHEALTH checklist for this trial.

### Participant Recruitment and Eligibility Criteria

Individuals were eligible if they met the following inclusion criteria: (1) aged 18 years or older, (2) high risk for diabetes, as measured by the American Diabetes Association (ADA) screening tool (score of 5 or more) [24], (3) access to WeChat push notifications with a smartphone, and (4) agreement to informed consent and further participation in the study. Exclusion criteria for participants were (1) diagnosis of diabetes or other endocrine diseases (eg, thyroid disease under treatment), (2) mobility impairment, (3) diet therapy due to physical illness, (4) uncontrolled hypertension (systolic blood pressure ≥160 mmHg, diastolic blood pressure ≥110 mmHg), (5) participation in a lifestyle intervention program or similar study or a plan to take medicine or undergo surgery to lose weight, or (6) pregnancy. Elimination criteria for participants were withdrawal from the study voluntarily or failure to complete the follow-up due to an illness.

Participants (n=81) who met the inclusion criteria were recruited from May to June 2018 in the physical examination center of Peking Union Medical College Hospital and were randomly allocated to the intervention group (social media intervention) or control group (printed material intervention) using a randomization list generated by a computerized program. The enrolled participants were linked to the online baseline questionnaires on site after providing signed informed consent and voluntarily completing the baseline survey. They were given a detailed explanation by researchers to help them understand the questions and the study better, as well as a brief (10-minute) explanation that they were at high risk for T2DM and that improved dietary behaviors and physical activity could help them delay or prevent the incidence. This in-person interaction allowed related web links for further data collection and interventions to be received by participants immediately; the subsequent procedure could be accessible to enrolled participants later. Due to the characteristics and design of this study, participants were not blinded to group assignment; data analysts, however, were blinded to the assignment.

### Study Setting and Data Collection

The enrolled participants were informed of group assignment by phone, text messages, or emails after leaving the study site. Both groups were provided with the same culturally tailored educational materials to encourage lifestyle modification, modified from the US DPP design plan and other evidence-based strategies [25]. TTM-based behavioral intervention techniques and strategies were applied in the delivery of the interventions to both groups. Participants in the mobile-based intervention group were told to follow the WeChat subscription account to access the details of the intervention program, including push notifications for health education content and links to online self-report questionnaires. Participants in the control group were mailed a lifestyle modification handbook. There was no direct interaction between participants in the different groups after randomization, and participants had no further contact with research staff except for reminders for the 3-month and 6-month online follow-up assessments. Reminder notifications for assessments were standardized across groups in content, delivery, and frequency to avoid bias from interactional differences. All follow-up assessment data were collected via WeChat applets connected to an encrypted database. Logic checks were conducted to identify suspicious submissions, and the suspected data errors were dropped from the collections and re-collected after participants understood the meaning of the questions with assistance from the research team members. To enhance the adherence and engagement of participants, each participant was compensated with office supplies valued at around ¥70 (US $10.50) per assessment.

### Study Conditions

#### The DHealthBar Intervention

DHealthBar provided 6 months of a WeChat-based behavior intervention aimed at improving eating habits and physical activity, thus reducing diabetes risk in the long term. It also aimed to increase the intention to change these behaviors. The 6-month mobile-based intervention was composed of educational material sent by the WeChat subscription account named DHealthBar, WeChat applets (lightweight apps that form part of the WeChat ecosystem, which could be used independently) embedded with online questionnaires, and a check-in applet serving as an online forum with functions similar to Twitter Moments.

**Figure 1:**
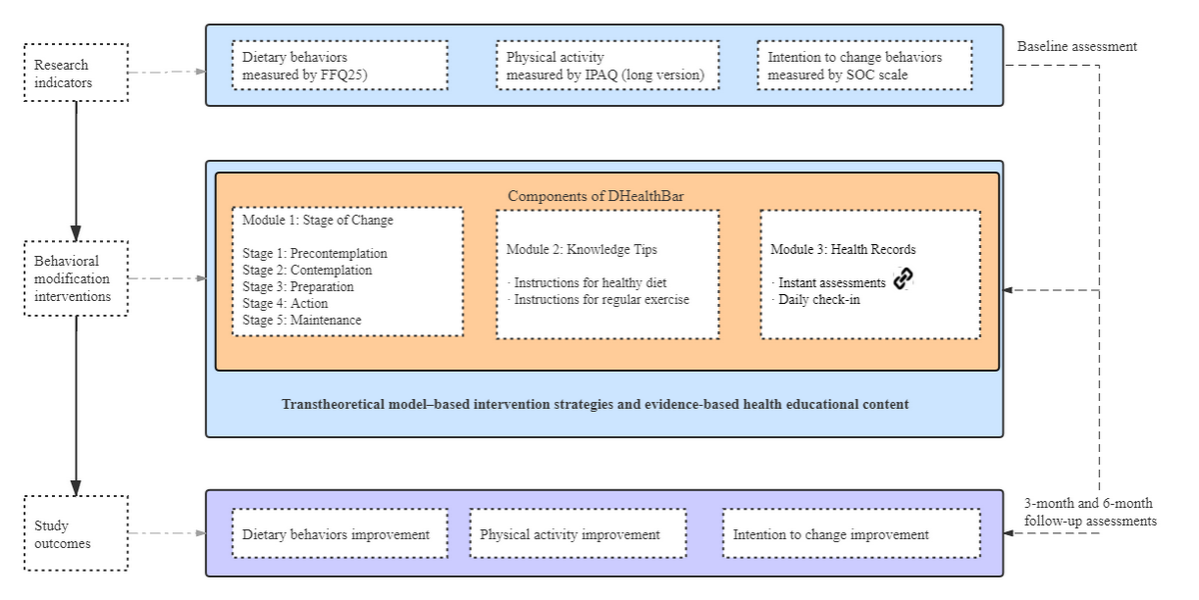
The schema of the TTM-based social media behavior intervention plan. FFQ25: food frequency questionnaire 25; IPAQ: International Physical Activity Questionnaire; SOC: Stage of Change; TTM: transtheoretical model.

The general design of DHealthBar and supporting details are displayed in Figure 1. It was designed to educate people at high risk for T2DM about diabetes prevention and specifically focus on providing practical strategies on relevant aspects, such as (1) interventions on behavior change, (2) behavior change instructions, (3) behavior change tracking tools (ie, online questionnaires), and (4) a common space for communication and sharing. Within the WeChat account, 3 modules were set to categorize the push messages: stages of change, knowledge tips, and health records (Figure 1). In the stages of change section, researchers created message interventions for the research participants to improve their motivation for behavior change (ie, from precontemplation to maintenance). The knowledge tips section provided practical instructions for participants to change their dietary behaviors and physical activity. In the health records section, the food frequency questionnaire 25 (FFQ25) diet-tracking tool [26], the International Physical Activity Questionnaire (IPAQ) physical activity tracking tool (long version in Chinese) [27], and the Stage of Change (SOC) Scale stages of change tracking tool [28] were presented in the instant assessment embedded in the WeChat applets and allowed immediate feedback upon submission. Furthermore, there was a check-in online forum for participants to record and share daily diet and exercises in the form of photos and texts; participants could appreciate and comment on tweets that interested them to encourage peers indirectly.

With the support of the DPP design plan [25] and localized evidence-based guidelines [29,30], the push messages were designed and enhanced with TTM-based [31] and evidence-based social media lifestyle intervention strategies [10,32,33] to achieve behavior change goals.

The educational content was designed following several principles. For eating behaviors, the focus was on decreasing added sugars and refined carbohydrates, decreasing saturated and trans fats, and increasing fruits and vegetables. Changes in food types and reduction in portion size were emphasized as means of reducing energy intake rather than specific calorie targets or calorie counting. For dietary behaviors, changing food types and reducing portion size, for instance, limiting the intake of sweetened beverages and refined carbohydrates, increasing fruits and vegetables, and replacing saturated and trans fats with polyunsaturated fats, was meant to (1) reduce total energy intake (in kcal per day) according to age, gender, and level of daily activities [30] and (2) modify the proportion of 3 macronutrients, with carbohydrates at 50% to 65%, protein at 10% to 15%, and fats at 20% to 30% [30].

For physical activity, participants were encouraged to enhance the endurance and frequency of their physical activity to achieve the goal of at least 30 minutes of moderate-intensity physical activity per day [25,29]. Resistance training was also encouraged.

The participant’s psychological readiness to take action for these behavioral changes was addressed using TTM-based behavior change techniques, specifically following strategies aimed at behavior change at different stages [17,28]. For instructions on behavior change skills, the educational material was arranged into comics, stories, or short articles and then sent as push messages on the WeChat subscription. The participants could leave messages about topics of interest and share the recommended content as their WeChat Moments. All presented information was designed with input from an endocrinologist, a diabetes nurse educator, registered dietitians, and health behavior specialists and was reviewed by them before release. Educational content about dietary behaviors and physical activity were posted for all engaging participants twice a week (Multimedia Appendix 2).

**Figure 2:**
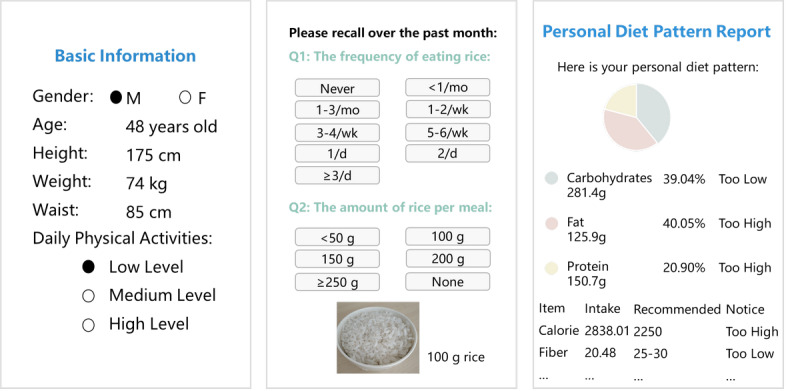
Screenshots of the online assessment for dietary behaviors. From left to right: the entry screen for basic information, the food frequency questionnaire 25, and the feedback information on personal dietary behaviors.

WeChat mini applets embedded with online questionnaires were used as automated trackers to encourage participants to monitor their daily health behavior and increase engagement (Figure 2). After completing the assessments, participants received automated feedback and personalized recommendations immediately. In particular, the processes of behavior change were used as indicators for tailored interventions for the participant’s intention to change and as a general guide for the behavior promotion intervention modifications in the next study period [31]. Due to the dynamics of intentions and lifestyle changes, an interval of 3 months was set to reevaluate the participants and rearrange the intervention domains and skills accordingly.

#### Alternative Care: Self-Learning With DPP-Based Handbook

The alternative care for participants in the control group was a behavior change handbook with instructions on dietary behaviors and physical activity modification. A follow-up parallel to the intervention group was conducted after 3 months and 6 months with online questionnaires embedded in the WeChat applets. Participants in the control group were informed of the assessment link at baseline assessment and received reminders through phone calls, text messages, or emails around the follow-up assessment time points. Apart from these time points, participants in the control group had no contact with researchers during the study period.

### Outcome Measures

The primary outcome measures were changes in dietary behaviors and physical activity at the 3-month and 6-month follow-up from baseline, serving as the key goals of our interventions. Secondary outcome measures were (1) stage of change for dietary behaviors and physical activity, which reflects one’s intention for behavior change, and (2) anthropometric characteristics, including BMI and waist circumference. Dietary behaviors were described using daily intake of energy; the macronutrients protein, carbohydrates, and fat; and macronutrient proportions. Physical activity was described using weekly physical activity in total and weekly physical activity of light, moderate, and vigorous intensity. Participants completed self-reported assessments at 3 time points: baseline, 3 months, and 6 months.

#### General Information

General information was collected during the screening process, including gender, age, body mass, body mass index, waist circumference, family history of diabetes, occupation, and education.

#### Diabetes Risk Screening

Diabetes self-screening tools published by the ADA for risk of type 2 diabetes [24] were adopted and included 6 measures—gender, age, family history, hypertension history, BMI, and sedentariness—with a total of 10 points. Scoring less than 5 points indicated a low risk of diabetes and scoring 5 or more points indicated the state of being prediabetic or at a high risk for diabetes.

#### Changes in Dietary Behaviors and Physical Activity

Changes in dietary behaviors and physical activity were evaluated to examine the effect of the interventions. The simplified FFQ25 developed by Gao et al [26] was adopted for the dietary behavior assessment. There are 25 types of staple foods and nonstaple foods included. The frequency and weight of food intake were recorded for calculation and analysis. The frequency of food intake was on a 9-point scale, ranging from “never eat” to “more than 3 times a day,” with a corresponding weight sequence of 0, 0.03, 0.07, 0.22, 0.50, 0.79, 1, 2, and 3. The amount of food intake was on a 6-point scale, ranging from “never eat” to “more than 3 times a day,” with a corresponding weight sequence of 0, 0.5, 0.75, 1, 1.5, and 2. The FFQ25 has been demonstrated to be a diet assessment tool with high reliability and validity. Results for dietary behaviors included energy intake (in kcal) per day, 3 macronutrient (ie, carbohydrates, fat, and protein) intakes (in g) per day, and macronutrient proportions (percentage), with higher values indicating greater intake of certain types of nutrients or corresponding types of food. Physical activity was assessed using the self-administered IPAQ (long version in Chinese) [27]. The IPAQ contains a total of 27 questions covering a comprehensive set of 4 domains of physical activity (ie, leisure time physical activity, domestic and gardening (yard) activities, work-related physical activity, and transport-related physical activity), with 2 added questions on sedentary behavior. In addition, items in the form were structured to provide details about the specific types of walking (*W*), moderate-intensity activity (*M*), and vigorous-intensity activity (*V*) within each domain. The volume of activity measured by the IPAQ can be weighted by metabolic equivalents of task (METs) that represent the energy required to yield a score in MET minutes. With selected MET values of each type, the MET score was calculated as the specific MET value (*W*, *M*, *V*) × weekly frequency (days per week) × time per day (minutes per day). In this study, because of the equivalent intensity, we used light-intensity activity (*L*) to replace walking activity (*W*) as one of our outcomes. With the weekly light-, moderate-, and vigorous-intensity physical activity summed up, we could determine the MET score for the weekly total physical activity.

In addition to the standardized approach, we adopted long-form IPAQ data processing rules to perform data cleaning and set an upper and lower threshold for the duration of activity, thus qualifying the results within a reasonable range [27].

#### Stage of Behavior Change

The SOC Scale, derived from the SOC Scale for reducing dietary fat intake used by Greene and Rossi [28], was adopted to evaluate the intention to change dietary behaviors and physical activity. The Cronbach α coefficient for each item was no less than .76. The stage assessment was divided into 5 phases based on the answers from the participants (precontemplation: “Do not intend to make dietary/physical activity changes in the next 6 months”; contemplation: “Intend to make dietary/physical activity changes in the next 6 months”; preparation: “Intend to make dietary/physical activity changes in the next 30 days”; action: “Have been changing dietary/physical activity but it has been less than 6 months“; maintenance: ”Have been changing dietary/physical activity for more than 6 months“).

### Statistics Analysis

Based on established methods [34,35], it was estimated that a sample of 74 participants would be required to detect a 715 kcal change in daily energy intake from baseline to 6 months with a standard deviation of 300 kcal, using an α level of .05 and a power level of 80%. The final desired estimated number of participants enrollment was set at 78 after 5% estimated attrition. Descriptive statistics were used to examine the baseline information, using number and percentage for nominal categorical variables and mean and standard deviation or median and interquartile range for normally or not normally distributed variables, respectively. Comparisons between groups at all 3 assessment points (baseline, 3 months, 6 months) were made on demographic measures (baseline only) and all outcome measures (ie, dietary behaviors, physical activity, stage of behavior change, and anthropometric characteristics). We used 2-tailed *t* tests for continuous variables that were normally distributed. Kruskal-Wallis tests were used for ordinal variables or continuous variables not normally distributed. Chi-square tests were used for nominal categorical variables.

To analyze the repeated outcome measures [36,37], generalized linear mixed models (GLMMs) were used to examine changes over time and differences between the 2 arms in dietary behaviors, physical activity, stage of behavior change, and anthropometric characteristics, which allowed response variables from different distributions, such as nonnormal distribution responses and ordinal categorical responses. Given both fixed and random effects, the general form of the model is [38]:

***y*=**β***X+uZ*+ε**

In the above equation, *y* is the outcome variable, *X* is a predictor variable, **β** is a fixed-effects regression coefficient, and *uZ* is designed for random effects, with *Z* being the random complement to the fixed *X* and *u* being the random complement to the fixed **β**. In addition, ε is designed for the residuals, the part of *y* that is not explained by the model.

The following form of the model is given to make this more concrete in this study setting:

***y*=**β**_1 _*X_1_*+*β_2 _ X_2_*+***β**_3 _ X_1 _ X_2_*+*Z*+ε**

In the above equation, *y* is the outcome variable, either continuous (ie, dietary behavior outcome measures, physical activity outcome measures, BMI, and waist circumference) or categorical (ie, stage of change for dietary behaviors or physical activity). **β**_1_*X*_1_ + **β**_2_*X*_2_ + **β**_3_*X*_1_*X*_2_ represents fixed effects. Further, in this study, variables were included for intervention (*X*_1_: 0=no, 1=yes) and time (*X*_2_: 1=baseline, 2=3-month follow-up, 3=6-month follow-up), and **β**s are fixed-effects regression coefficients. Particularly, to interpret the unique effect of the intervention on time, an intervention-time interaction item (**β**_3_*X*_1_*X*_2_) was added. *Z*, the random intercept, is incorporated for the random effects of participants. All analyses were adjusted for baseline age, gender, education level, and occupational level for the probability of an impact on outcome measures [39,40]. Outcomes of GLMM analyses were reported as exponentiated coefficients (exp[**β**]). Adjusted *P* values and confidence intervals to control the type I error rate at 0.05 (95% CI) were computed through simulations (Multimedia Appendices 3 and 4). Specific model type, link function, and the total number of observations included in the model are listed in the footnotes of Multimedia Appendices 3 and 4.

We conducted post hoc comparisons of the intervention group versus the control group at the 3-month follow-up, the intervention group versus the control group at the 6-month follow-up, the difference in mean changes or proportion changes between the intervention group and the control group at the 3-month and 6-month follow-up, and within-group comparisons between each time point (ie, 0 months vs 3 months, 0 months vs 6 months, 3 months vs 6 months). The first 2 post hoc comparisons evaluated whether the mean levels of outcome measures in the intervention group differed from those of the control group at each time point. The middle 2 comparisons evaluated whether there was a change in the differences between the treatment group and the control group over time. The last comparisons examined the changes in outcome measures across the study period. Besides these comparisons, for the main-effects-only model, if the intervention was statistically significant, post hoc comparisons were conducted to determine if outcome measures differed significantly in mean or proportion between the 2 groups.

All the tests were 2-tailed and used a .05 significance level. All analyses were conducted using per-protocol analysis with R (version 3.6.2; R Foundation for Statistical Computing).

## Results

### Overview

**Figure 3:**
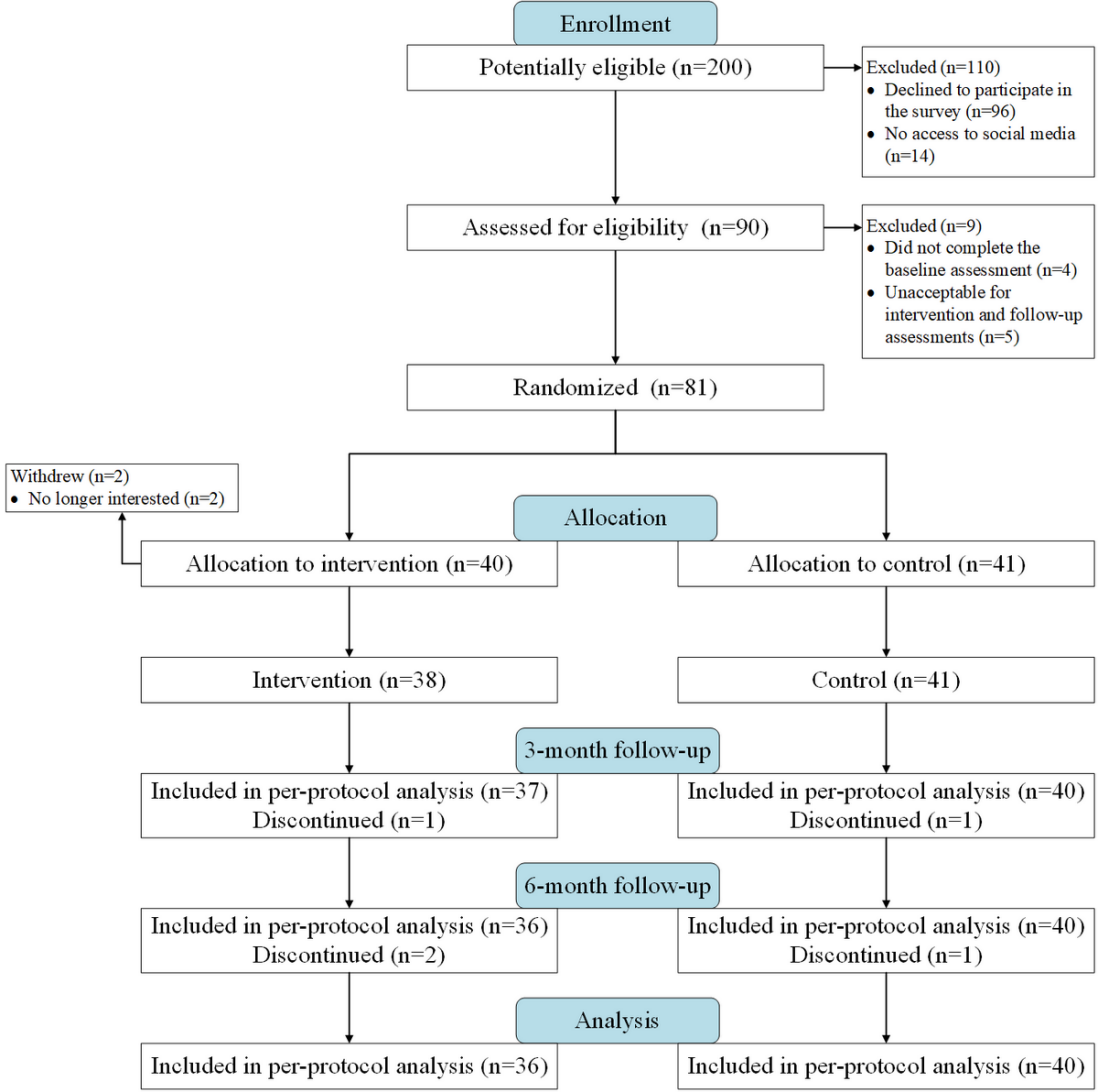
Flow diagram for the randomized trial.

A pragmatic, parallel-group, randomized controlled trial in Beijing, China, was conducted from May 2018 to March 2019. Of the 200 potentially eligible participants, 90 (45.0%) agreed to a further test. After excluding 7 participants who declined to participate further (2 after the informed group assignment) and 4 who provided invalid questionnaires, a total of 79 of the 90 (88%) participants were randomly allocated to the social media intervention group (n=38) or control group (n=41). There was high retention and participation in follow-up assessments; of the 79 participants, 78 (99%) were followed up with at 3 months and 76 (96%) were followed up with at 6 months. Analyses of the attrition rate did not show statistically significant difference between the 2 groups (*χ*²_1_*=*0.0; *P*=.61). Hence, attrition was not systemic. Figure 3 shows the participants’ flow through the study.

The participants were middle-aged (control group: median 47.5, IQR 44.0-55.0 years; intervention group: 46.0, IQR 44.0-48.2 years) and well educated, with 51 of the 76 participants (67%) having a college degree or higher. At baseline, most participants were at relatively low stages (ie, precontemplation, contemplation, or preparation) of dietary behavior or physical activity change. Baseline equivalence of participants was demonstrated between the 2 groups in sociodemographic, anthropometric, and behavioral characteristics (Table 1). No participants in the study did not complete the follow-up assessment for the reason of being diagnosed with diabetes, nor did anyone report adverse events caused by the social media intervention.

**Table 1:** Characteristics and baseline behaviors of participants in the DHealthBar trial.

Characteristics				Control (n=40)		Intervention (n=36)		*t* test^a^ (*df*)		H value^b^ (*df*)		Chi-square test (*df*)		*P* value
Age (years), median (IQR)				47.5 (44.0-55.0)		46.0 (44.0-48.2)		N/A^c^		2.03 (1)		N/A		.15
**Gender, n (%)**								N/A		N/A		0.4^d^ (1)		.52
	Male			24 (60.0）		18 (50.0）								
	Female			16 (40.0）		18 (50.0）								
Body mass (kg), median (IQR)				70.0 (65.0-80.5)		72.7 (65.0-80.0)		N/A		0.55 (1)		N/A		.46
BMI (kg/m²), median (IQR)				24.7 (23.4-26.1)		25.3 (24.7-26.2)		N/A		2.75 (1)		N/A		.10
Waist circumference (cm), median (IQR)				81.0 (73.7-85.0)		82.5 (77.7-88.0)		N/A		1.48 (1)		N/A		.22
**Family history of diabetes, n (%)**								N/A		N/A		1.7^d^ (1)		.19
	Yes			13 (32.5)		18 (50.0）								
	No			27 (67.5)		18 (50.0）								
**Occupational classification, n (%)**								N/A		N/A		1.9^e^ (9)		.99
	Administrator or manager			3 (7.5)		2 (5.5)								
	Office and administrative support			12 (30.0)		10 (28.0)								
	Health care practitioner			2 (5.0)		2 (5.5)								
	Legal profession			3 (7.5)		2 (5.5)								
	Architect or engineer			3 (7.5)		5 (14.0)								
	Laborer or protective service worker			4 (10.0)		4 (11.1)								
	Driver			3 (7.5)		2 (5.5)								
	Teacher			4 (10.0)		4 (11.1)								
	Computer programmer			2 (5.0)		3 (8.3)								
	Retired			4 (10.0)		2 (5.5)								
**Education level, n (%)**								N/A		N/A		0.9^e^ (2)		.72
	Primary			1 (2.5)		1 (2.8)								
	Secondary			14 (35.0)		9 (25.0)								
	Tertiary			25 (62.5)		26 (72.2)								
**Dietary behaviors (FFQ25^f^)**														
	Energy intake (kcal/d), median (IQR)			2029.0 (1515.0-2547.0)		2263.0 (2047.0-2419.0)		N/A		2.12 (1)		N/A		.14
	**Macronutrients** **intake (g/d), median (IQR)**													
		Fat	78.3 (56.9-103.2)		81.2 (30.9-38.3)		N/A		0.001 (1)		N/A		.97	
		Carbohydrates	210.7 (141.3-272.4)		228.8 (153.2-256.3)		N/A		0.29 (1)		N/A		.59	
		Protein	94.7 (80.4-128.5)		98.3 (63.2-123.9)		N/A		0.78 (1)		N/A		.38	
	**Macronutrients** **proportion (%)**													
		Fat, median (IQR)	37.3 (33.0-40.9)		36.8 (30.9-38.3)		N/A		1.18 (1)		N/A		.28	
		Carbohydrates, mean (SD)	42.7 (7.2)		44.6 (6.2)		1.22 (73.88)		N/A		N/A		.23	
		Protein, mean (SD)	20.8 (2.3)		20.1 (2.4)		–1.20 (72.03)		N/A		N/A		.23	
**Physical activity (IPAQ ^g^), median MET^h^/wk (IQR)**														
	Total			2632.5 (1577.7-3746.2)		2385.5 (803.2-3852.0)		N/A		0.81 (1)		N/A		.37
	Light intensity			891.0 (334.1-1980.0)		627.0 (457.9-1485.0)		N/A		0.49 (1)		N/A		.48
	Moderate intensity			1230.5 (612.5-2520.5)		720.0 (315.0-1590.0)		N/A		2.08 (1)		N/A		.15
	Vigorous intensity			0.0 (0.0-495.0)		0.0 (0.0-740.0)		N/A		0.8 (1)		N/A		.37
**Stage of dietary behaviors change (SOC^i^** **), n (%)**								N/A		0.14 (1)		N/A		.71
	Precontemplation			13 (32.5)		7 (19.4)								
	Contemplation			15 (37.5)		20 (55.6)								
	Preparation			6 (15.0)		6 (16.7)								
	Action			3 (7.5)		1 (2.8)								
	Maintenance			3 (7.5)		2 (5.5)								
**Stage of physical activity change (SOC), n (%)**								N/A		0.02 (1)		N/A		.89
	Precontemplation			7 (17.5)		6 (16.7)								
	Contemplation			22 (55.0)		19 (52.8)								
	Preparation			5 (12.5)		6 (16.7)								
	Action			4 (10.0)		5 (13.8)								
	Maintenance			2 (5.0)		0 (0.0)								

^a^2-sample, 2-tailed *t* test.

^b^Kruskal-Wallis test.

^c^N/A: not applicable.

^d^Chi-squared test.

^e^Fisher exact test.

^f^FFQ25: simplified food frequency questionnaire 25.

^g^IPAQ: International Physical Activity Questionnaire (long version in Chinese).

^h^MET: metabolic equivalent of task.

^i^SOC: Stage of Change.

### Primary Outcomes: Dietary Behaviors and Physical Activity Changes

The results of the outcome measures and univariate analyses at the 3- and 6-month follow-up assessments are reported in Multimedia Appendix 5. For outcome measures in dietary behaviors, mean levels of energy intake (*P*<.001), macronutrient intake of fat and protein (fat: *P*<.001; protein: *P*<.001), and proportion of fat (*P*<.001) were significantly lower in the intervention group than in the control group at the 6-month follow-up assessment, while the proportion of carbohydrates was significantly higher than that in the control group (*P*<.001) (Multimedia Appendix 5).

Significant group × time interaction effects and significant main effects for time were both observed in intake of energy and the 3 macronutrients, as well as in total physical activity and moderate-intensity physical activity per week (Table 2 and Figure S1 in Multimedia Appendix 6). Significant main effects for time were also observed in light-intensity physical activity per week. Significant main effects for the intervention were only observed in the intake of protein and carbohydrate proportions (Table 2).

For dietary behaviors, energy intake decreased significantly at 3 months and 6 months compared with baseline in both groups (control: 3 months, *P*<.001; 6 months, *P*=.003; intervention: 3 months, *P*<.001; 6 months, *P*<.001). See Multimedia Appendix 3 for exp(**β**) and 95% CI values. The intake of the 3 macronutrients decreased significantly at 6 months compared with baseline in both groups (control: fat, *P*=.047; carbohydrates, *P*=.01; protein, *P*=.006; intervention: fat, *P*<.001; carbohydrates, *P*<.001; protein, *P*=.001). Intake of fat (*P*=.049) and protein (*P*<.001) decreased significantly at 3 months compared with baseline in the control group. A significant decrease in fat proportion was observed in the intervention group at 6 months (*P*=.01). For physical activity, total physical activity and light-intensity physical activity per week improved significantly at 6 months compared with baseline in both groups (control: total, *P*=.03; light-intensity physical activity, *P*=.01; intervention: total, *P<*.001; light-intensity physical activity, *P<*.001). There were continuously significant changes in energy intake (decrease) and moderate-intensity physical activity (increase) in the intervention group (3 vs 0 months: energy, *P*<.001; moderate-intensity physical activity, *P*=.04; 6 vs 3 months: energy, *P*<.001; moderate-intensity physical activity, *P*=.03). *P* values in this paragraph were adjusted.

At 6 months, the intake of energy (*P*<.001), fat (*P*<.001), and protein (*P*=.003) and the intake proportion of fat (*P*=.003) were significantly lower in the intervention group than in the control group, while the proportion of carbohydrate intake (*P*=.03) was significantly higher in the intervention group than in the control group. See Multimedia Appendix 4 for exp(**β**) and 95% CI values. Significantly larger decreases over 6 months were observed in the intervention group in energy (*P*<.001), fat (*P*<.001), and carbohydrate (*P*<.001) intakes compared with the control group. In addition, the increase in moderate-intensity physical activity was significantly larger in the intervention group than in the control group (*P*=.002). *P* values in this paragraph were adjusted.

**Table 2:** The effect of the intervention and time on primary and secondary outcomes over the intervention period.

Outcome measures			Model effects					
			Group		Time		Group × time	
			*F* test (*df*)	*P* value	*F* test (*df*)	*P* value	*F* test (*df*)	*P* value
**Dietary behaviors (FFQ25^a^)**								
	Energy intake^b^		1.07 (1,70)	.30	73.30 (2,148)	<.001^c^	27.59 (2,148)	<.001^c^
	**Macronutrient intake^b^**							
		Fat	3.23 (1,70)	.08	23.26 (2,148)	<.001^c^	7.07 (2,148)	.001^c^
		Carbohydrates	0.08 (1,70)	.78	21.78 (2,148)	<.001^c^	4.58 (2,148)	.01^c^
		Protein	4.83 (1,70)	.03^c^	24.73 (2,148)	<.001^c^	5.88 (2,148)	.003^c^
	**Macronutrients proportion (%)**							
		Fat^b^	3.91 (1,70)	.05	2.01 (2,148)	.14	2.56 (2,148)	.08
		Carbohydrates^b^	4.75 (1,70)	.03^c^	1.90 (2,148)	.15	1.65 (2,148)	.19
		Protein^d^	2.67 (1,70)	.11	1.76 (2,148)	.17	0.16 (2,148)	.85
**Physical activity (IPAQ^e^)**								
	Total^d^		0.04 (1,70)	.84	19.38 (2,148)	<.001^c^	3.68 (2,148)	.03^c^
	Light intensity^f^		0.39 (1,66.83)	.53	19.42 (2,133)	<.001^j^	1.77 (2,133)	.17
	Moderate intensity^g^		0.16 (1,66.65)	.69	9.63 (2,139.7)	.001^c^	6.84 (2,139.7)	.001^c^
	Vigorous intensity^h^		3.87 (1,50.53)	.05	0.08 (2,76.37)	.92	0.51 (2,76.07)	.60
Stage of dietary behavior change (SOC^i,j^)			3.56 (1,145)	.06	6.37 (2,145)	.002^c^	27.18 (2,145)	<.001^c^
Stage of physical activity change (SOC^i^)			2.66 (1,145)	.10	6.31 (2,145)	.002^c^	47.76 (2,145)	<.001^c^
**Anthropometric characteristics^b^**								
	BMI (kg/m²)		0.47 (1,70)	.50	125.40 (2,148)	<.001^c^	21.68 (2,148)	<.001^c^
	Waist circumference (cm)		4.54 (1,70)	.04^c^	172.47 (2,148)	<.001^c^	44.62 (2,148)	<.001^c^

^a^FFQ25: simplified food frequency questionnaire 25.

^b^Model (response variable of gamma distribution with log link) included age, gender, education level, and occupational classification as covariates. Number of observations=228.

^c^*P* values represent statistically significant results, *P*<.05.

^d^Model (response variable of lognormal distribution with identity link) included age, gender, education level, and occupational classification as covariates. Number of observations=228.

^e^IPAQ: International Physical Activity Questionnaire (long version in Chinese).

^f^Model (response variable of lognormal distribution with identity link) included age, gender, education level, and occupational classification as covariates. Number of observations=213.

^g^Model (response variable of lognormal distribution with identity link) included age, gender, education level, and occupational classification as covariates. Number of observations=220.

^h^Model (response variable of lognormal distribution with identity link) included age, gender, education level, and occupational classification as covariates. Number of observations=132.

^i^Model (response variable of multinomial distribution with cumulative logit link) included age, gender, education level, and occupational classification as covariates. Number of observations=228.

^j^SOC: Stage of Change.

### Secondary Outcomes: Stage of Behavior Change and Anthropometric Characteristics

According to univariate analyses (Multimedia Appendix 5), there was a statistically significant difference between the 2 groups in the stage of change for dietary behaviors and physical activity at 6 months *(P*<.001) (Multimedia Appendix 5). A statistically significant difference between the 2 groups in the stage of change for dietary behaviors was also observed at 3 months (*P*=.02) (Multimedia Appendix 5).

Significant group × time interaction effects and significant main effects for time were both observed at 6 months in the stage of change for dietary behaviors and physical activity, as well as in anthropometric characteristics (ie, BMI and waist circumference) (Table 2 and Figure S2 in Multimedia Appendix 6). Significant main effects of the intervention were only observed in anthropometric characteristics (Table 2).

There were continuous, statistically significant changes in all secondary outcome measures in the intervention group (3 vs 0 months: diet SOC, *P*<.001; physical activity SOC, *P*<.001; BMI, *P*<.001; waist, *P*<.001; 6 vs 3 months: diet SOC, *P*<.001, physical activity SOC, *P*<.001; BMI, *P*<.001; waist, *P*<.001). See Multimedia Appendix 3 for exp(**β**) and 95% CI values. Statistically significant changes in all secondary outcome measures were observed in the control group at 6 months (diet SOC: *P*<.001; physical activity SOC: *P*<.001; BMI: *P*<.001; waist: *P*<.001). *P* values in this paragraph were adjusted.

There were significant between-group differences in the distribution of the stages of change for dietary behaviors and physical activity at 6 months; participants in the intervention group had a higher probability of being at higher stages for these behavior changes (diet SOC: *P*=.002; physical activity SOC: *P*=.01). See Multimedia Appendix 4 for exp(**β**) and 95% CI values. Significantly greater improvements were observed at 6 months in the probability of being at a higher stage of change in dietary behaviors (*P*=.002) and physical activity (*P*=.003). There was also a significantly greater decrease in anthropometric characteristics in the intervention group (BMI: *P*<.001; waist: *P*<.001). Additionally, the decrease in waist circumference was significantly greater in the intervention group than in the control group at 3 months (*P*<.001). Adjusted *P* values were used in this paragraph.

## Discussion

### Principal Findings

This study examined the effectiveness of DHealthBar in improving diet behaviors and physical activity for people at high risk for T2DM. TTM-based behavioral change techniques were applied during the research. Our findings show that this WeChat applet could be effective in inducing positive dietary changes and improving physical activity within a 6-month intervention period. Significant decreases in energy and macronutrient intake were found in both groups, but these decreases differed substantially between groups in energy, fat, and carbohydrate intake. In addition, considerable improvements over 6 months in total and light-intensity physical activity were found in both groups, with no substantial between-group differences. This social media intervention also showed positive effects on participants’ intentions to change these behaviors (measured using stages of behavior change) and anthropometric characteristics.

These improved outcomes are comparable to those of other mobile-based intervention studies that have shown favorable results in improving dietary behaviors and physical activity [33,40-42]. Regardless of the limitations of self-reported assessments, it was encouraging to find a continuous change in dietary behaviors and physical activity associated with the social media intervention. Furthermore, a high level of user engagement and retention was achieved within 6 months. We owe these benefits partly to the merits of the WeChat platform, which is wide reaching, cost-efficient, and easy to use. Shared by disparate populations, WeChat enables health educators to present complex content in the form of vivid graphics or videos, thus spreading health education content for the public in a feasible and accessible way [43]. Given the heavy burden resulting from the growing prevalence of T2DM and the fact that high-risk populations with sedentary lifestyles and dietary patterns rich in fat and refined carbohydrates are much more likely to develop diabetes and other chronic diseases associated with unhealthy lifestyles [1,4], it is plausible to adopt mobile-based approaches to facilitate the provision of behavioral interventions and increase the uptake with good scalability [5,44]. As such, our findings might be insightful for population-level delivery of diet change and physical activity improvement techniques for diabetes prevention, given the wide reach of social media interventions [5,43].

The significant decreases in daily energy and macronutrient intakes in both groups indicated that interventions incorporating behavior change theory and techniques tend to be effective. This observation was supported by previous randomized studies based on TTM behavior intervention strategies [19,20,41]. Changes in dietary macronutrient patterns were examined in this study, and a significant decrease in the proportion of fat intake was observed in the intervention group. Previous studies targeting dietary change have focused on the reduction of energy intake or the consumption of certain foods, such as vegetables, fruits, and low-fat milk [40,41,45]. Interestingly, to the best of our knowledge, few studies have focused on the effect of mHealth interventions on changes in macronutrient patterns. Therefore, this finding might be useful, as it suggests the potential of mHealth interventions to modify dietary macronutrient patterns, which have proved critical to weight loss and cardiovascular risk management [46].

Designed as an extension of DPP-based lifestyle interventions, DHealthBar provided the participants in the intervention group with behavioral support after baseline that was delivered through social media, whereas those in the control group underwent a completely self-guided behavior modification. There would be an enhancement in the efficacy of mHealth or eHealth interventions if they were provided with behavioral support or guidance from counselors either online or face to face, with an insight shared in conclusions related to diabetes prevention and other areas [47]. Consistent with this, a continuous improvement in dietary behaviors and physical activity was observed. In the control group, however, considerable heterogeneity existed in the effects over time, and the participants tended to relapse or stagnate after an immediate improvement (<3 months). DHealthBar, which was delivered through a social media platform, provided interaction between care providers and receivers. Since the push notifications with lifestyle instructions were available to all the participants in the intervention group, participants could comment below them. Researchers could be informed of the participant’s preferences and requests for educational content and improve the content accordingly as soon as possible.

Aligned with traditional mHealth interventions delivered through text messages, websites, or smartphone apps [33,40,42,48], behavior change techniques, including self-monitoring, goal setting, and tailored educational content and feedback, may have contributed to the effectiveness of the dietary change and physical activity intervention in this study. As a result of the self-report tools embedded in the WeChat platform, individuals could receive timely automated feedback to better understand their stage achievements and current status [48,49], thereby enhancing their engagement. In addition, some of the effects may have been due to the strengths inherent in social media, especially the sense of interpersonal connection, which is underlined as a key factor of the success of mHealth programs [50]. In the DHealthBar trial, a sense of community was created. Participants in the intervention arm were connected via a social network, where clustered peers concerned about the same health issue provided social and emotional support to each other in an online forum [11,41]. Additionally, they were encouraged to track their daily routines of food or exercise and post them as “moments” to receive appreciation and comments from others.

The positive effects of the social media intervention on physical activity were modest. A significant difference between the 2 groups, however, was observed in moderate-intensity physical activity improvement after 6 months, which is partly ascribed to sociodemographic factors. Most participants in this study were middle-aged and had relatively high socioeconomic indicators. The physical activity of middle-aged or older adults shares a strong pattern of high frequency and slow pace [51]. Accordingly, the exercise program recommended in this study tended to include items such as brisk walking, tai chi, and Chinese square dancing, whereas vigorous physical activity and muscle strength training were only mentioned occasionally. Despite the evidence that adults at higher socioeconomic levels are more likely to show greater engagement in physical activity, due to their improved health-related knowledge and skills [39,52], the type of activity was limited.

One of the strengths of this study was the theory-driven nature of the study design, which allowed the procedures of the intervention and evaluation to be relatively empirical. TTM-based behavioral intervention techniques sensitively measured the stages of change that participants underwent and precisely identified the fluctuations in participants’ intentions to change behaviors that were affected by dynamical barriers or facilitators. Hierarchical strategies could therefore be used according to the readiness of individuals [53]. Additionally, the progression of participants’ behavior improvement in this study kept pace with participants’ stage of change level. One explanation is that substantial change in actual behavior might partly be ascribed to interventions concerned with cognition and intention modification, which serve as critical mediators of actual behavioral change [54]. As previously claimed, the degree of motivation among participants is likely to influence results [52,55].

In this study, changes in anthropometric markers (ie, BMI and waist circumference decreases) were additional benefits in the context of an intensive behavior modification trial. This finding corresponds with the results from similar studies [41,56,57]. To maintain relatively low monetary and time costs, we included physiological measures, such as BMI and waist circumference, but not biochemical markers of physiological health. However, given the genetic and somatotype characteristics of diabetes in East Asia [58,59], as well as the probable independence of glycemic benefit from weight loss or behavior modification [40,41], it is plausible to further include biochemical markers associated with glycemic improvements to induce more indicative clinical outcomes [60].

We acknowledge several limitations of this study. First, our sample is limited in terms of both size and geographical coverage, which might attenuate the power and generalizability of our study. Second, due to initial financial resource limitations and the desire to maintain relatively low attrition by assuring participants’ active engagement within a reasonable period, this randomized trial lasted for only 6 months. Third, participants were not blinded to randomization; consequently, we could not exclude the social desirability bias, that is, whether the postassessments or stand-alone interventions acted as incentives to the participants to provide a positive response. Fourth, due to considerations of cost-effectiveness and convenience, self-reported questionnaires were used for data collection in this study [39], which achieved the additional goal of avoiding the potential burden resulting from wearable tracking tools [45]. Given the characteristics of our assessment tools, which had high reliability and validity, and the characteristics of our participants, who had high education levels, the confounding effect might be insignificant [61]. However, there is no denying that recall bias did exist, which may have resulted in an erroneous estimation of the association between behavior changes and interventions, as mentioned by previous studies [61,62]. Last, despite the strong evidence base for the TTM, substantial heterogeneity remains in the effects of health behavior interventions based on this model, which have not been confirmed uniformly [31,63].

In this study, we demonstrated the initial effect of DHealthBar, a mobile behavior intervention coupled with theory-driven instructions, on individuals recruited from a single region. Investigators in future studies should assess the effectiveness in more diverse socioeconomic contexts. In addition, the usage of this social media platform could be measured to obtain more insights into the participants’ perceptions of the intervention program. Furthermore, novel measurement tools could be included in the future to objectively measure the outcomes, under the condition of such tools being affordable and acceptable. Analyses of images and text on social media with health behavior tags might help health professionals obtain a better understanding of the modification procedure that the participants underwent and reveal potential clinical influences [64,65]. Future work should elucidate the “active ingredients” of the techniques, as well as effective ways to employ them [66], thus shedding light on the design of mHealth interventions.

### Conclusion

DHealthBar was effective in promoting dietary behaviors and physical activity among populations at high risk for T2DM. Our findings, coupled with high engagement and retention, may suggest the strength of social media as a health behavior intervention delivery mode, inspiring further work on diet and physical activity promotion.

## Appendix

Multimedia Appendix 1 CONSORT EHEALTH checklist (V 1.6.1).

Multimedia Appendix 2 TTM-based behavioral intervention strategies and contents.

Multimedia Appendix 3 Within-group comparisons of outcomes over the intervention period.

Multimedia Appendix 4 Between-group comparisons of outcomes over the intervention period.

Multimedia Appendix 5 Results for outcome measures at 3-month and 6-months follow-up assessments and univariate analyses.

Multimedia Appendix 6 Interaction plots of outcome measures.

## Abbreviations

ADA: American Diabetes Association
DPP: Diabetes Prevention Program
FFQ25: food frequency questionnaire 25
GLMM: generalized linear mixed model
IPAQ: International Physical Activity Questionnaire
MET: metabolic equivalent of task
mHealth: mobile health
SOC: Stage of Change
T2DM: type 2 diabetes mellitus
TTM: transtheoretical model
