# Supplementary material for: A Mobile-Based Intervention for Dietary Behavior and Physical Activity Change in Individuals at High Risk for Type 2 Diabetes Mellitus: Randomized Controlled Trial
Source: JMIR Mhealth Uhealth. 2020 Nov 3;8(11):e19869. doi: 10.2196/19869 (PMC7671838; doi:10.2196/19869)
Supplement: Multimedia Appendix 2 [file mhealth_v8i11e19869_app2.pdf]

Multimedia Appendix 2. TTM-based behavioral intervention strategies and contents

Table 1. Internet behavioral intervention stages and contents

Stages of Change	Intervention Contents
Precontemplation	The definition, diagnosis, risk factors, adverse effects, treatment, and prevention of type 2 diabetes and prediabetes<br>The relationship of type 2 diabetes and prediabetes<br>The relationship of behaviors and type 2 diabetes<br>The benefits of behavior change
Contemplation	The pros and cons: consequences and conditions of behavior change<br>Coping strategies for averse conditions
Preparation	Health behavior goals and alternative action methods targeting each goal<br>Strategies for how to seek help or supports
Action	Alternative replacement behaviors<br>Strategies for overcoming problematic environmental aspects<br>Common “drawbacks” and solutions<br>Tips for behavior changing actions
Maintenance	Common “drawbacks” and solutions<br>Tips for behavior maintenance<br>Strategies for adjusting negative emotions

Table 2. Social media behavior intervention stages and strategies

Stages of Change	Goals	Techniques/Strategies of Processes of Change	Techniques/Strategies of Decisional Balance	Techniques/Strategies of Self-efficacy	Social media Techniques or Supports
Precontemplation	Individuals can understand that unhealthy behaviors bring direct and indirect harm and the relationship between unhealthy behaviors and type 2 diabetes.	Consciousness raising: (1) Arousing the consciousness of behavior change: provide information about harm of unhealthy behavior; help each individual work out the importance of behavior change and then generate the consciousness of behavior change through diabetes risk assessment. (2) Cognitive restructuring：helping individuals analyze the reasons for unhealthy behaviors and recognize the benefits of behavior change.<br>Dramatic relief: encouraging individuals to release negative emotions by telling stories of their experiences with others.<br>Environmental reevaluation：role modeling, e.g., vignette depicting parents provide good examples by healthy eating for children.	Providing information about the effects of behavior change on disease prevention;<br>Improving the consciousness of the benefits of behavior change.	N/A	Online evaluation;<br>Graphics, text, and (or) audio;<br>Social media forums or online discussion
Contemplation	Individuals list and then weigh the pros and cons of changing.	Self-reevaluation: (1) Identifying ambivalence if the intention to change but not ready to take action; learning individuals' perception during behavior change and providing adequate support. (2) Encouraging reflection about unhealthy behaviors, e.g., guided imagery exercise for helping understand the health consequences of overeating.	Encouraging individuals to list their pros and cons of behavior changing and weighing them.	N/A	Online evaluation;<br>Graphics, text, and (or) audio;
Preparation	Individuals can recognize problems and obstacles while changing behavior.	Self-liberation: helping identify a diet and exercise goal and developing a 1 week menu and action plan for reaching the goal.	Measuring and estimating problems and obstacles of behavior change and providing resolution strategies and skills for related problems and obstacles.	Behavior goal setting;<br>Duly promising;<br>Seeking support from families or friends.	Online evaluation;<br>Graphics, text, and (or) audio;
Action	Individuals can properly deal with problems they face during the process of	Counterconditioning: interactive activity for helping individuals identify problem behaviors that prevent them from maintaining diet and exercise followed by a multimedia presentation of healthy replacement behaviors;	The same as in preparation, especially focusing on providing strategies to handle environmental temptation, e.g. eating out;	Discussing the feasibility of the goal and action plan with;<br>Encouraging individuals to try to change behavior;	Online evaluation;<br>Graphics, text, and (or) audio;<br>Social media forums or online discussion

	behavior change.	Help relationships: development of a “buddy contract” for teaming up on line with someone with interests of achieving similar behavior goals;<br>Reinforcement management: strengthening proper diet or exercise behavior, e.g., encouraging improvement of behavior change and giving positive feedback;<br>Stimulus control: environmental assessment activity for determining characteristics of home, neighborhood, and school environments that may encourage unhealthy eating and exercise, followed by a discussion of relevant change strategies.	For those with previous experiences of failure, exploring reasons with follow up supportive advice.	Providing positive reinforcement of individuals’ improvement of behavior changing.	
Maintenance	Individuals can maintain behavior and prevent relapse.	N/A	Evaluating individuals’ behavior and adjust coping methods against difficulties during behavior change;<br>Commitment to individuals' while improving behavior change.	Maintaining effective communication;<br>Observing behavior via diaries and giving encouragement to strengthen new behavior;<br>Adjusting individuals’ negative emotions.	Online evaluation;<br>Graphics, text, and (or) audio;<br>Social media forums or online discussion
