# Supplementary material for: A Mobile-Based Intervention for Dietary Behavior and Physical Activity Change in Individuals at High Risk for Type 2 Diabetes Mellitus: Randomized Controlled Trial
Source: JMIR Mhealth Uhealth. 2020 Nov 3;8(11):e19869. doi: 10.2196/19869 (PMC7671838; doi:10.2196/19869)
Supplement: Multimedia Appendix 3 [file mhealth_v8i11e19869_app3.pdf]

Multimedia Appendix 3. Within-group comparisons of outcomes over the intervention period.

Outcome measures	exp(β)（95%CI）	*t*, adj.*P*	exp(β)（95%CI）	*t*, adj.*P*
	Control(n=40)		Intervention(n=36)	
**Dietary behaviors, FFQ 25[a]**				
Energy intake[b]				
3 vs 0[c]	0.83(0.74-0.92)	*t*=-4.65, adj.*P*<.001[k]	0.76(0.68-0.85)	*t*=-6.49, adj.*P*<.001[k]
6 vs 0[c]	0.87(0.78-0.96)	*t*=-3.52, adj.*P*=.003[k]	0.57(0.51-0.64)	*t*=-13.06, adj.*P*<.001[k]
6 vs 3[c]	1.05(0.94-1.16)	*t*=1.13, adj.*P*=.74	0.76(0.68-0.85)	*t*=-6.57, adj.*P*<.001[k]
**Macronutrients intake[b]**				
Fat				
3 vs 0[c]	0.83(0.68-1.00)	*t*=-2.62, adj.*P*=.049[k]	0.84(0.68-1.02)	*t*=-2.31, adj.*P*=.11
6 vs 0[c]	0.82(0.68-0.99)	*t*=-2.64, adj.*P*=.047[k]	0.59(0.48-0.72)	*t*=-6.90, adj.*P*<.001[k]
6 vs 3[c]	1.00(0.82-1.21)	*t*=-0.01, adj.*P*=.99	0.70(0.57-0.86)	*t*=-4.59, adj.*P*<.001[k]
Carbohydrate				
3 vs 0[c]	0.84(0.72-0.97)	*t*=-3.13, adj.*P*=.01[k]	0.88(0.75-1.03)	*t*=-2.20, adj.*P*=.14
6 vs 0[c]	0.84(0.72-0.97)	*t*=-3.11, adj.*P*=.01[k]	0.69(0.59-0.81)	*t*=-6.12, adj.*P*<.001[k]
6 vs 3[c]	1.00(0.86-1.16)	*t*=0.02, adj.*P*=1.00	0.79(0.67-0.92)	*t*=-3.92, adj.*P*<.001[k]
Protein				
3 vs 0[c]	0.78(0.66-0.92)	*t*=-3.85, adj.*P*<.001[k]	0.92(0.77-1.09)	*t*=-1.31, adj.*P*=.62
6 vs 0[c]	0.78(0.66-0.92)	*t*=-3.85, adj.*P*=.006[k]	0.67(0.56-0.80)	*t*=-6.04, adj.*P*<.001[k]
6 vs 3[c]	1.00(0.85-1.18)	*t*=-0.00, adj.*P*=1.00	0.73(0.61-0.87)	*t*=-4.73, adj.*P*=.001[k]
**Macronutrients proportion**				
Fat[b]				
3 vs 0[c]	0.98(0.90-1.08)	*t*=-0.48, adj.*P*=.98	1.00(0.90-1.10)	*t*=-0.10, adj.*P*=1.00
6 vs 0[c]	1.00(0.91-1.09)	*t*=-0.06, adj.*P*=1.00	0.91(0.82-1.00)	*t*=-2.57, adj.*P*=.01[k]
6 vs 3[c]	1.01(0.93-1.11)	*t*=0.42, adj.*P*=.99	0.91(0.83-1.00)	*t*=-2.46, adj.*P*=.08

Outcome measures	exp(β)（95%CI） Control(n=40)	t, adj.P	exp(β)（95%CI） Intervention(n=36)	t, adj.P
Carbohydrate[b]				
3 vs 0[c]	1.02(0.93-1.12)	t=0.56, adj.P=.97	1.00(0.91-1.11)	t=0.14, adj.P=1.00
6 vs 0[c]	1.01(0.92-1.11)	t=0.34, adj.P=1.00	1.09(0.99-1.20)	t=2.27, adj.P=.12
6 vs 3[c]	1.00(0.91-1.09)	t=-0.22, adj.P=1.00	1.08(0.98-1.19)	t=2.13, adj.P=.17
Protein[d]				
3 vs 0[c]	0.99(0.92-1.06)	t=-0.53, adj.P=.98	0.99(0.92-1.07)	t=-0.40, adj.P=.99
6 vs 0[c]	0.97(0.92-1.05)	t=-1.01, adj.P=.81	0.95(0.88-1.03)	t=-1.59, adj.P=.44
6 vs 3[c]	0.99(0.92-1.06)	t=-0.49, adj.P=.98	0.97(0.89-1.04)	t=-1.19, adj.P=.71
**Physical activity, IPAQ[e]**				
Total[d]				
3 vs 0[c]	1.07(0.83-1.37)	t=0.69, adj.P=.95	1.48(1.14-1.93)	t=3.92, adj.P<.001[k]
6 vs 0[c]	1.31(1.02-1.68)	t=2.83, adj.P=.03[k]	1.80(1.39-2.35)	t=5.89, adj.P<.001[k]
6 vs 3[c]	1.23(0.95-1.57)	t=2.14, adj.P=.16	1.22(0.94-1.58)	t=1.97, adj.P=.23
Light-intensity[f]				
3 vs 0[c]	1.22(0.92-1.61)	t=1.87, adj.P=.28	1.50(1.13-1.99)	t=3.82, adj.P=.001[k]
6 vs 0[c]	1.38(1.05-1.82)	t=3.08, adj.P=.01[k]	1.80(1.37-2.38)	t=5.62, adj.P<.001[k]
6 vs 3[c]	1.13(0.86-1.49)	t=1.20, adj.P=.70	1.20(0.91-1.58)	t=1.78, adj.P=.32
Moderate-intensity[g]				
3 vs 0[c]	0.97(0.68-1.39)	t=-0.22, adj.P=1.00	1.48(1.02-2.16)	t=2.76, adj.P=.04[k]
6 vs 0[c]	1.07(0.75-1.53)	t=0.50, adj.P=.98	2.20(1.51-3.19)	t=5.56, adj.P<.001[k]
6 vs 3[c]	1.10(0.77-1.57)	t=0.72, adj.P=.94	1.48(1.02-2.14)	t=2.80, adj.P=.03[k]
Vigorous-intensity[h]				
3 vs 0[c]	1.15(0.76-1.76)	t=0.91, adj.P=.87	0.95(0.63-1.43)	t=-0.34, adj.P=1.00
6 vs 0[c]	1.06(0.69-1.61)	t=0.35, adj.P=1.00	1.01(0.67-1.52)	t=0.04, adj.P=1.00
6 vs 3[c]	0.91(0.64-1.30)	t=-0.68, adj.P=.95	1.06(0.75-1.50)	t=0.46, adj.P=.99

Outcome measures	exp(β)（95%CI） Control(n=40)	*t*, adj.*P*	exp(β)（95%CI） Intervention(n=36)	*t*, adj.*P*
**Stage of behavior change, SOC[i,j]**				
Dietary behaviors				
3 vs 0[c]	4.83(0.81-28.76)	*t*=2.29, adj.*P*=.10	19.62(5.11-75.27)	*t*=5.73, adj.*P*<.001[k]
6 vs 0[c]	20.03(4.58-87.60)	*t*=4.02, adj.*P*<.001[k]	464.62(96.77-2230.80)	*t*=7.74, adj.*P*<.001[k]
6 vs 3[c]	4.14(0.96-17.92)	*t*=2.52, adj.*P*=.06	23.69(5.10-71.94)	*t*=5.63, adj.*P*<.001[k]
Physical activity				
3 vs 0[c]	2.64(0.55-12.67)	*t*=1.63, adj.*P*=.41	11.86(4.36-32.28)	*t*=4.88, adj.*P*<.001[k]
6 vs 0[c]	10.65(3.96-28.64)	*t*=4.73, adj.*P*<.001[k]	230.67(61.83-860.52)	*t*=8.17, adj.*P*<.001[k]
6 vs 3[c]	4.03(1.32-12.33)	*t*=2.47, adj.*P*=.07	19.45(5.99-63.12)	*t*=4.98, adj.*P*<.001[k]
**Anthropometric characteristics[b]**				
BMI				
3 vs 0[c]	0.98(0.96-0.99)	*t*=-5.00, adj.*P*=<.001[k]	0.96(0.95-0.97)	*t*=-8.06, adj.*P*<.001[k]
6 vs 0[c]	0.97(0.95-0.98)	*t*=-6.71, adj.*P*<.001[k]	0.92(0.91-0.94)	*t*=-15.34, adj.*P*<.001[k]
6 vs 3[c]	0.99(0.98-1.00)	*t*=-1.71, adj.*P*=.37	0.96(0.95-0.98)	*t*=-7.29, adj.*P*<.001[k]
Waist circumference				
3 vs 0[c]	0.99(0.98-1.00)	*t*=-3.92, adj.*P*=.001[k]	0.97(0.96-0.98)	*t*=-10.59, adj.*P*<.001[k]
6 vs 0[c]	0.98(0.98-0.99)	*t*=-6.60, adj.*P*<.001[k]	0.95(0.94-0.95)	*t*=-19.28, adj.*P*<.001[k]
6 vs 3[c]	0.99(0.99-1.00)	*t*=-2.68, adj.*P*=.46	0.98(0.97-0.98)	*t*=-8.69, adj.*P*<.001[k]

[a]FFQ 25: Simplified Food Frequency Questionnaire 25

[b]Model (response variable of gamma distribution with log link) included age, gender, education level, occupational classification as covariates. Number of observations=228

[c]Reference category for comparison.

[d]Model (response variable of lognormal distribution with identity link) included age, gender, education level, occupational classification as covariates. Number of observations=228

[e]IPAQ: International Physical Activity Questionnaire -long (Chinese version)

[f]Model (response variable of lognormal distribution with identity link) included age, gender, education level, occupational classification as covariates. Number of observations=213

[g]Model (response variable of lognormal distribution with identity link) included age, gender, education level, and occupational classification as covariates. Number of observations=220

[h]Model (response variable of lognormal distribution with identity link) included age, gender, education level, and occupational classification as covariates. Number of observations=132

[i]Model (response variable of multinomial distribution with cumulative logit link) included age, gender, education level, and occupational classification as covariates. Number of observations=228.

[j]SOC: Stage of change scale.

[k]Adjusted *P* values represent statistically significant results, adj. $P<.05$.
