# Supplementary material for: A Mobile-Based Intervention for Dietary Behavior and Physical Activity Change in Individuals at High Risk for Type 2 Diabetes Mellitus: Randomized Controlled Trial
Source: JMIR Mhealth Uhealth. 2020 Nov 3;8(11):e19869. doi: 10.2196/19869 (PMC7671838; doi:10.2196/19869)
Supplement: Multimedia Appendix 4 [file mhealth_v8i11e19869_app4.pdf]

Multimedia Appendix 4. Between-group comparisons of outcomes over the intervention period.

Outcome measures	exp(β) (95%CI)	t, adj.P	exp(β) (95%CI)	t, adj.P	exp(β) (95%CI)	t, adj.P
	3 months		6 months		Overall	
**Dietary behaviors, FFQ 25[a]**						
Energy intake[b]						
Intervention VS Control[c]	1.02(0.86-1.21)	*t*=0.34,adj.*P*=1.00	0.74(0.62-0.88)	*t*=-4.69,adj.*P*<.001[k]	0.94(0.81-1.09)	*t*=-1.04,adj.*P*=.84
Difference in mean change	0.91(0.78-1.07)	*t*=-1.51,adj.*P*=.53	0.66(0.56-0.77)	*t*=-7.05,adj.P<.001[k]		
**Macronutrients intake[b]**						
Fat						
Intervention VS Control[c]	1.00(0.79-1.28)	*t*=0.02,adj.*P*=1.00	0.70(0.55-0.90)	*t*=-3.87,adj.*P*<.001[k]	0.89(0.74-1.06)	*t*=-1.80,adj.*P*=0.35
Difference in mean change	1.01(0.76-1.35)	*t*=0.13,adj.*P*=1.00	0.71(0.54-0.95)	*t*=-3.19,adj.*P*<.001[k]		
Carbohydrate						
Intervention VS Control[c]	1.08(0.86-1.35)	*t*=0.91,adj.*P*=.90	0.85(0.68-1.06)	*t*=-1.94,adj.*P*=.26	0.98(0.82-1.18)	*t*=-0.28,adj.*P*=1.00
Difference in mean change	1.05(0.84-1.31)	*t*=0.56,adj.*P*=.98	0.83(0.66-1.03)	*t*=-2.30,adj.*P*<.001[k]		
Protein						
Intervention VS Control[c]	1.03(0.83-1.27)	*t*=0.37,adj.*P*=1.00	0.75(0.61-0.93)	*t*=-3.63,adj.*P*=.003[k]	0.88(0.75-1.03)	*t*=-2.20,adj.*P*=.16
Difference in mean change	1.17(0.91-1.49)	*t*=1.70,adj.*P*=.40	0.85(0.67-1.09)	*t*=-1.73,adj.*P*=.38		
**Macronutrients proportion**						
Fat[b]						
Intervention VS Control[c]	0.99(0.88-1.10)	*t*=-0.36,adj.*P*=1.00	0.89(0.79-0.99)	*t*=-2.99,adj.*P*<.001[k]	0.95(0.88-1.02)	*t*=-1.98,adj.*P*=.25
Difference in mean change	1.01(0.88-1.16)	*t*=0.26,adj.*P*=1.00	0.91(0.79-1.05)	*t*=-1.82,adj.*P*=.33		
Carbohydrate[b]						
Intervention VS Control[c]	1.02(0.92-1.14)	*t*=0.69,adj.*P*=.49	1.12(1.00-1.25)	*t*=2.80,adj.*P*=.03[k]	1.06(0.99-1.14)	*t*=2.18,adj.*P*=.16
Difference in mean change	0.99(0.86-1.13)	*t*=-0.28,adj.*P*=1.00	1.07(0.94-1.23)	*t*=1.41,adj.*P*=.60		
Protein[d]						
Intervention VS Control[c]	0.97(0.89-1.06)	*t*=-0.88,adj.*P*=.92	0.95(0.87-1.04)	*t*=-1.54,adj.*P*=.52	0.96(0.91-1.02)	*t*=-1.63,adj.*P*=.45
Difference in mean change	1.00(0.90-1.11)	*t*=0.07,adj.*P*=1.00	0.98(0.88-1.09)	*t*=-0.46,adj.*P*=1.00		

Outcome measures	exp(β)（95%CI）	*t*, adj.*P*	exp(β)（95%CI）	*t*, adj.*P*	exp(β)（95%CI）	*t*, adj.*P*
	3 months		6 months		Overall	
**Physical activity IPAQ[e]**						
Total[d]						
Intervention VS Control[c]	1.08(0.68-1.73)	*t*=0.45,adj.*P*=.99	1.07(0.67-1.71)	*t*=0.41,adj.*P*=1.00	0.97(0.64-1.47)	*t*=-0.21,adj.*P*=1.00
Difference in mean change	1.39(0.96-2.00)	*t*=2.37,adj.*P*=.10	1.38(0.96-1.99)	*t*=2.33,adj.*P*=.11		
Light-intensity[f]						
Intervention VS Control[c]	0.93(0.54-1.61)	*t*=-0.33,adj.*P*=1.00	0.99(0.58-1.70)	*t*=-0.04,adj.*P*=1.00	0.89(0.54-1.46)	*t*=-0.63,adj.*P*=.98
Difference in mean change	1.23(0.83-1.83)	*t*=1.38,adj.*P*=.61	1.31(0.88-1.93)	*t*=1.81,adj.*P*=.33		
Moderate-intensity[g]						
Intervention VS Control[c]	0.97(0.54-1.73)	*t*=-0.15,adj.*P*=1.00	1.30(0.73-2.31)	*t*=1.20,adj.*P*=.74	0.93(0.56-1.52)	*t*=-0.40,adj.*P*=1.00
Difference in mean change	1.53(0.91-2.57)	*t*=2.16,adj.*P*=.16	2.05(1.23-3.44)	*t*=3.68,adj.*P*=.002[k]		
Vigorous-intensity[h]						
Intervention VS Control[c]	1.34(0.72-2.50)	*t*=1.25,adj.*P*=.69	1.55(0.85-2.85)	*t*=1.95,adj.*P*=.26	1.50(0.86-2.62)	*t*=1.97,adj.*P*=.25
Difference in mean change	0.82(0.45-1.49)	*t*=-0.89,adj.*P*=.90	0.95(0.52-1.73)	*t*=-0.22,adj.*P*=1.00		
**Stage of behavior change, SOC[i,j]**						
Dietary behaviors						
Intervention VS Control[c]	4.69(0.71-31.04)	*t*=1.62,adj.*P*=.42	26.80(3.51-204.91)	*t*=3.20,adj.*P*=.002[k]	5.26(0.92-29.89)	*t*=1.89,adj.*P*=.27
Difference in proportion change	4.06(0.95-17.32)	*t*=1.91,adj.*P*=.26	23.19(4.06-132.28)	*t*=3.57,adj.*P*=.002[k]		
Physical activity						
Intervention VS Control[c]	3.24(0.55-18.99)	*t*=1.31,adj.*P*=.66	15.60(2.67-91.04)	*t*=3.08,adj.*P*=.01[k]	3.31(0.77-14.16)	*t*=1.63,adj.*P*=.43
Difference in proportion change	4.49(0.92-21.87)	*t*=1.88,adj.*P*=.29	21.65(3.91-119.89)	*t*=3.55,adj.*P*=.003[k]		

Outcome measures	exp(β) (95%CI)	t, adj.*P*	exp(β) (95%CI)	t, adj.*P*	exp(β) (95%CI)	t, adj.*P*
	3 months		6 months		Overall	
**Anthropometric characteristics[b]**						
BMI						
Intervention VS Control[c]	0.97(0.96-1.09)	*t*=0.84,adj.*P*=.91	0.99(0.93-1.05)	*t*=-0.38,adj.*P*=1.00	1.02(0.95-1.08)	*t*=0.68,adj.*P*=.96
Difference in mean change	0.98(0.96-1.00)	*t*=-2.40,adj.*P*=.09	0.95(0.93-0.97)	*t*=-6.51,adj.*P*<.001[k]		
Waist circumference						
Intervention VS Control[c]	1.04(0.99-1.09)	*t*=2.08,adj.*P*=.18	1.02(0.97-1.07)	*t*=1.14,adj.*P*=.75	1.04(0.99-1.09)	*t*=2.13,adj.*P*=.16
Difference in mean change	0.98(0.97-0.99)	*t*=-4.98,adj.*P*<.001[k]	0.96(0.95-0.97)	*t*=-9.44,adj.*P*<.001[k]		

[a]FFQ 25: Simplified Food Frequency Questionnaire 25

[b]Model (response variable of gamma distribution with log link) included age, gender, education level, occupational classification as covariates. Number of observations=228

[c]Reference category for comparison.

[d]Model (response variable of lognormal distribution with identity link) included age, gender, education level, occupational classification as covariates. Number of observations=228

[e]IPAQ: International Physical Activity Questionnaire -long (Chinese version)

[f]Model (response variable of lognormal distribution with identity link) included age, gender, education level, occupational classification as covariates. Number of observations=213

[g]Model (response variable of lognormal distribution with identity link) included age, gender, education level, and occupational classification as covariates. Number of observations=220

[h]Model (response variable of lognormal distribution with identity link) included age, gender, education level, and occupational classification as covariates. Number of observations=132

[i]Model (response variable of multinomial distribution with cumulative logit link) included age, gender, education level, and occupational classification as covariates. Number of observations=228.

[j]SOC: Stage of change scale.

[k]Adjusted *P* values represent statistically significant results, adj. *P*<.05.
