# Supplementary material for: A Mobile-Based Intervention for Dietary Behavior and Physical Activity Change in Individuals at High Risk for Type 2 Diabetes Mellitus: Randomized Controlled Trial
Source: JMIR Mhealth Uhealth. 2020 Nov 3;8(11):e19869. doi: 10.2196/19869 (PMC7671838; doi:10.2196/19869)
Supplement: Multimedia Appendix 5 [file mhealth_v8i11e19869_app5.pdf]

Multimedia Appendix 5. Results for outcome measures at 3-month and 6-months follow-up assessments and univariate analyses

Outcome measures	3-month follow-up				6-month follow-up			
	Control（n=40）	Intervention(n=36)	*T/H/χ²*	*P*	Control（n=40）	Intervention(n=36)	*T/H/χ²*	*P*
**Dietary behavior, FFQ 25[a]**								
Energy intake[b] (kcal/day), median(IQR)[b]	1165.0(1198.0-2045.0)	1687.3(1498.8-1924.4)	0.34	.56	1855.2(1491.6-2200.1)	1249.0(1169.0-1352.0)	18.96	<.001[f]
**Macronutrients intake (g)** [b]								
Fat, median(IQR)	62.4(44.4-83.9)	64.85(49.8-79.4)	0.13	.71	77.3(52.7-85.4)	43.3(35.0-53.4)	14.7	<.001[f]
Carbohydrate, median(IQR)	170.2(136.5-217.8)	188.3(162.8-222.0)	1.07	.30	176.9(141.8-227.2)	159.9(121.8-179.8)	3.1	.08
Protein, median(IQR)	80.0(62.1-100.2)	85.8(57.4-94.8)	0.06	.95	84.3(69.8-100.9)	59.7(52.8-67.3)	15.88	<.001[f]
**Macronutrients proportion (%)**								
Fat, mean (SD)[c]	35.9(7.5)	35.2(5.5)	-0.47	.63	36.5(5.6)	32.2(6.3)	-3.08	<.001[f]
Carbohydrate, mean (SD)[c]	43.5(8.5)	44.9(7.4)	0.72	.47	43.2(7.5)	48.5(8.4)	2.85	<.001[f]
Protein, median(IQR)[b]	20.0(18.6-23.3)	19.8(18.0-21.5)	-1.04	.30	20.11(18.3-22.59)	18.6(17.5-20.8)	3.31	.07
**Physical activity (MET/week) , median (IQR) , IPAQ[b,d]**								
Total	3096.00 (1892.8-5969.0)	3440.0(1669.0-4718.0)	0.07	.80	3120.0(2089.0-5168.0)	4226.0(1964.0-5663.0)	0.76	.38
Light-intensity	1089.0(445.5-2103.8)	1353.0(676.5-2004.2)	0.19	.66	1353.0(705.4-2202.8)	1386.0(792.0-2122.0)	0.04	.83
Moderate-intensity	1020.0(592.5-2100.0)	1200.0 (630.0-1770.0)	0.07	.79	1200.0(630.0-2505.0)	1510.0(1132.0-2040.0)	3.24	.07
Vigorous-intensity	300.0(0.0-712.5)	495.0 (0.0-866.2)	0.95	.33	270.0(0.0-760.0)	595.0(123.8-990.0)	3.05	.08
**Stage of dietary behaviors change, n (%), SOC[b,e]**								
Precontemplation	8(20.0)	1(2.8)	5.00	.02[f]	4(10.0)	0(0.0)	14.27	<.001[f]
Contemplation	12(30.0)	6(16.7)			11(27.5)	0(0.0)		
Preparation	11(27.5)	20(55.5)			8(20.0)	7(19.4)		
Action	5(12.5)	6(16.7)			13(32.5)	20(55.6)		
Maintenance	4(10.0)	3(8.3)			4(10.0)	9(25.0)		

Outcome measures	3-month follow-up<br>Control（n=40）	Intervention(n=36)	*T/H/χ²*	*P*	6-month follow-up<br>Control（n=40）	Intervention(n=36)	*T/H/χ²*	*P*
**Stage of physical activity change, n (%), SOC[b,e]**								
Precontemplation	7(17.5)	2(5.5)	2.74	.10	1(2.5)	0(0.0)	13.39	<.001[f]
Contemplation	11(27.5)	10(27.8)			14(35.0)	1(2.8)		
Preparation	13(32.5)	11(30.6)			11(27.5)	8(22.2)		
Action	7(17.5)	10(27.8)			9(22.5)	18(50.0)		
Maintenance	2(5.0)	3(8.3)			5(12.5)	9(25.0)		
**Anthropometric characteristics, median (IQR)[b]**								
BMI(kg/m²)	24.1(23.3-25.6)	24.1 (23.5-25.2)	0.03[f]	.85	24.2(22.8-25.6)	23.2(22.7-24.3)	2.42	.12
Waist circumference	80.0 (74.5-85.0)	80.0(75.9-85.5)	0.21	.64	78.2(8.0)	78.4(5.9)	-0.13	.89

[a]FFQ 25: Simplified Food Frequency Questionnaire 25.

[b]Kruskal–Wallis test, presented by *H* value, *P*-value.

[c]two-sample *t*-test, presented by *t* value, *P*-value.

[d]IPAQ: International Physical Activity Questionnaire -long (Chinese version).

[e]SOC: Stage of change scale

[f]*P* values represent statistically significant results, *P*<.05.
