# Supplementary material for: A Mobile-Based Intervention for Dietary Behavior and Physical Activity Change in Individuals at High Risk for Type 2 Diabetes Mellitus: Randomized Controlled Trial
Source: JMIR Mhealth Uhealth. 2020 Nov 3;8(11):e19869. doi: 10.2196/19869 (PMC7671838; doi:10.2196/19869)
Supplement: Multimedia Appendix 6 [file mhealth_v8i11e19869_app6.pdf]

Multimedia Appendix 6. **Interaction plots of outcome measures**

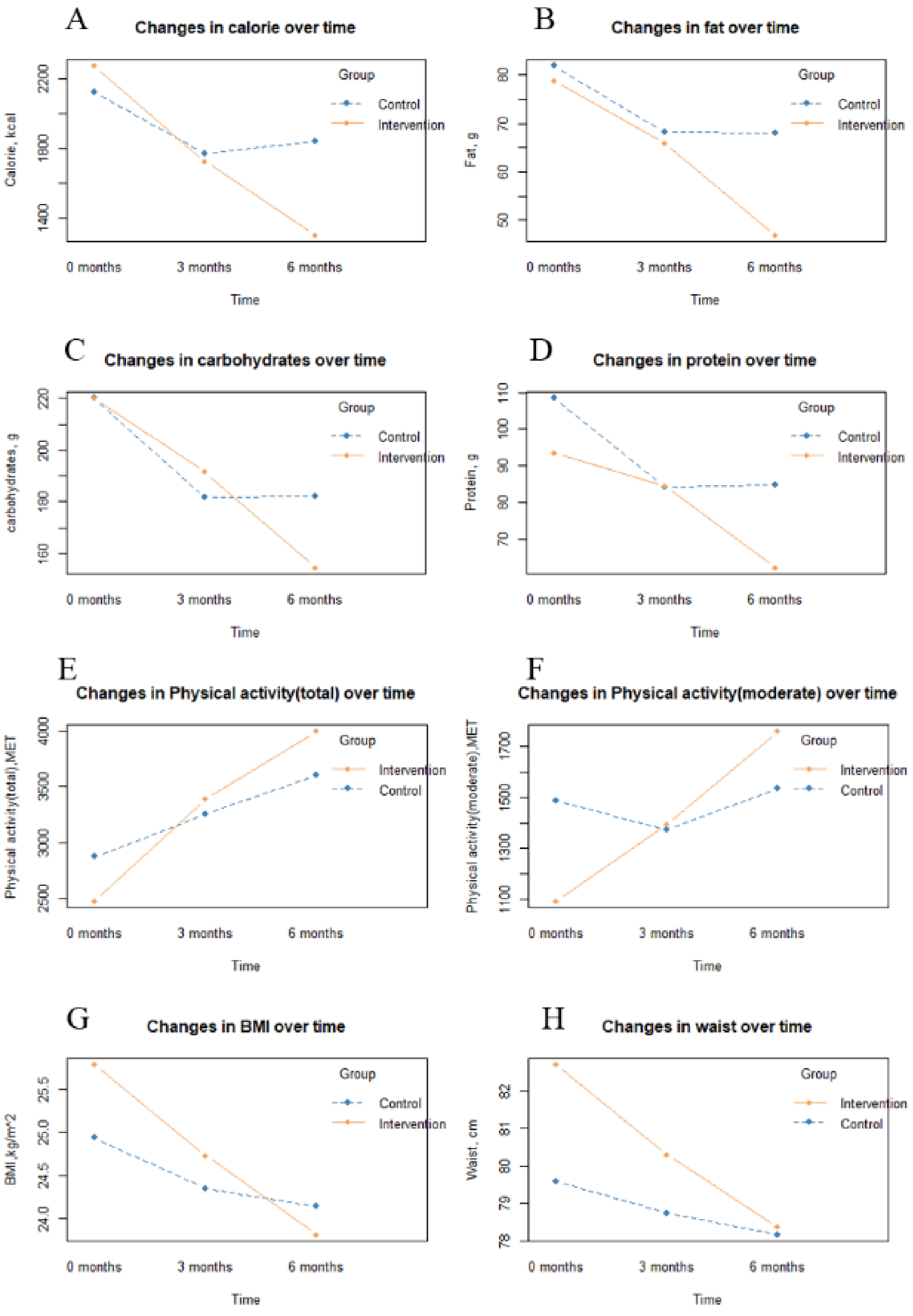

Figure S1. Changes of mean levels of outcome measures along with time.

Mean level change of outcome measures were represented in figure 1, where significant intervention-time-interaction effect was found.

A. Energy intake response (calorie) following the intervention. At 3-month follow-up the control group demonstrated at lower calorie intake levels compared to the intervention group, but became the higher one at 6-month follow-up.

B. Fat intake response following the intervention. Individuals that received the intervention demonstrated lower levels of fat intake compared to control group at 3-month and 6-month follow-up.

C. Carbohydrate intake response following the intervention. At 3-month follow-up the control group demonstrated lower Carbohydrates intake levels compared to the intervention group, but became the higher one at 6-month follow-up.

D. Protein intake response following the intervention. At 3-month follow-up the control group demonstrated lower Carbohydrates intake levels compared to the intervention group, but became the higher one at 6-month follow-up.

E. Total physical activity (weekly) response following the intervention. Individuals that received the intervention demonstrated higher levels of total physical activity (weekly) compared to control group at 3-month and 6-month follow-up.

F. Moderate-intensity physical activity (weekly) response following the intervention. Individuals that received the intervention demonstrated higher levels of moderate-intensity physical activity (weekly) compared to control group at 3-month and 6-month follow-up.

G. BMI response following the intervention. At 3-month follow-up the control group demonstrated lower Carbohydrates intake levels compared to the intervention group, but became the higher one at 6-month follow-up.

H. Waist circumstance response following the intervention. Individuals in control group demonstrated lower levels of fat intake compared to intervention group at 3-month and 6-month follow-up, the difference decreased at month 6 relative to month 3.

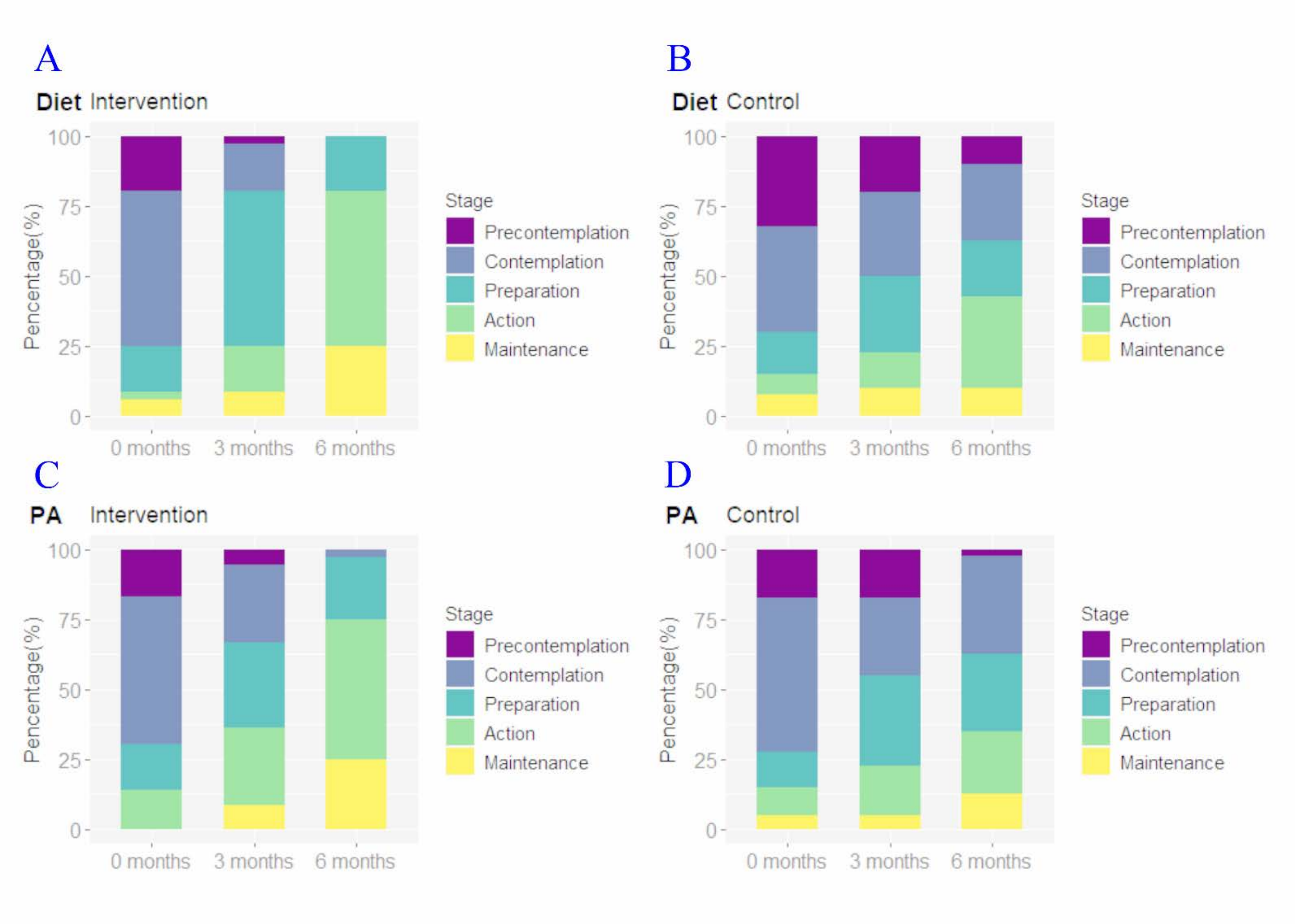

Figure S2. Changes of proportion of outcome measures along with time.

Changes of proportion of outcome measures were represented in figure 2, where significant intervention-time-interaction effect was found.

A. Stage of change for dietary behaviors in intervention group. There was a higher probability of being at higher stage of dietary behaviors changes along with the interventions last. 26 (78.8%) participants at relatively lower stage (ie, precontemplation, contemplation or preparation) of dietary behaviors modification at baseline, transferred to higher stage (ie, action or maintenance) after 6 months, with 29 (80.6)% individuals at relatively higher stage at that time point. The distribution of stage of change for dietary behaviors among individuals in the intervention group differed significantly between each time point.

B. Stage of change for dietary behaviors in control group. There was a higher probability of being at higher stage of dietary behaviors changes along with the interventions last. 11 (32.3%) participants at relatively lower stage (ie, precontemplation, contemplation or preparation) of dietary behaviors modification at baseline, transferred to higher stage (ie, action or maintenance) after 6 months, with 42.5% individuals at relatively higher stage of dietary behaviors change at that time point. Significant difference was observed at 6 months compared to baseline.

C. Stage of change for physical activity in intervention group. There was a higher probability of being at higher stage of physical activity changes along with the interventions last. 22 (71.0%) participants at relatively lower stage (ie, precontemplation, contemplation or preparation) of

physical activity modification at baseline, transferred to higher stage (ie, action or maintenance) after 6 months, with 27 (75.0%) individuals at relatively higher stage at that time point. The distribution of stage of changes for physical activity among individuals in the intervention group differed significantly between each time point.

B. Stage of change for physical activity in control group. There was a higher probability of being at higher stage of dietary behaviors changes along with the interventions last. 8 (23.5)% participants at relatively lower stage (ie, precontemplation, contemplation or preparation) of dietary behaviors modification at baseline, transferred to higher stage (ie, action or maintenance) after 6 months, with 35.0% individuals at relatively higher stage of dietary behaviors change at that time point. Significant difference was observed at 6 months compared to baseline.
